# Tubulointerstitial nephritis and uveitis syndrome and SARS-CoV-2 infection in an adolescent: just a coincidence in time?

**DOI:** 10.1007/s00467-023-05950-w

**Published:** 2023-05-02

**Authors:** Sonia García-Fernández, Eva Fernández-Morán, Cecilia López-Martínez, Blanca Vivanco-Allende, Carmen Costales-Álvarez, Flor A. Ordóñez-Álvarez

**Affiliations:** 1 Department of Pediatrics, Hospital Universitario Central de Asturias, Oviedo, Spain; 2 Instituto de Investigación Sanitaria del Principado de Asturias, Oviedo, Spain; 3 Centro de Investigación Biomédica En Red–Enfermedades Respiratorias, Instituto de Salud Carlos III, Madrid, Spain; 4 Department of Pathology, Hospital Universitario Central de Asturias, Oviedo, Spain; 5 Departamento de Cirugía y Especialidades Médico-Quirúrgicas, Área de Anatomía Patológica, Universidad de Oviedo, Oviedo, Spain; 6 Department of Ophthalmology, Hospital Universitario Central de Asturias, Oviedo, Spain

**Keywords:** TINU syndrome, SARS-CoV-2, Tubulointerstitial nephritis, Uveitis

## Abstract

**Background:**

Despite recent well-established kidney tropism of severe acute respiratory syndrome coronavirus 2 (SARS-CoV-2), usually presenting as acute kidney injury (AKI), there are few published cases with SARS-CoV-2-related tubulointerstitial nephritis (TIN). We report an adolescent with TIN and delayed association with uveitis (TINU syndrome), where SARS-CoV-2 spike protein was identified in kidney biopsy.

**Case-diagnosis/treatment:**

A 12-year-old girl was assessed for a mild elevation of serum creatinine detected during the evaluation of systemic manifestations including asthenia, anorexia, abdominal pain, vomiting, and weight loss. Data of incomplete proximal tubular dysfunction (hypophosphatemia and hypouricemia with inappropriate urinary losses, low molecular weight proteinuria, and glucosuria) were also associated. Symptoms had initiated after a febrile respiratory infection with no known infectious cause. After 8 weeks, the patient tested positive in PCR for SARS-CoV-2 (Omicron variant). A subsequent percutaneous kidney biopsy revealed TIN and immunofluorescence staining with confocal microscopy detected the presence of SARS-CoV-2 protein S within the kidney interstitium. Steroid therapy was started with gradual tapering. Ten months after onset of clinical manifestations, as serum creatinine remained slightly elevated and kidney ultrasound showed mild bilateral parenchymal cortical thinning, a second percutaneous kidney biopsy was performed, without demonstrating acute inflammation or chronic changes, but SARS-CoV-2 protein S within the kidney tissue was again detected. At that moment, simultaneous routine ophthalmological examination revealed an asymptomatic bilateral anterior uveitis.

**Conclusions:**

We present a patient who was found to have SARS-CoV-2 in kidney tissue several weeks following onset of TINU syndrome. Although simultaneous infection by SARS-CoV-2 could not be demonstrated at onset of symptoms, since no other etiological cause was identified, we hypothesize that SARS-CoV-2 might have been involved in triggering the patient’s illness.

## Introduction

Tubulointerstitial nephritis (TIN) and uveitis (TINU syndrome) are rare conditions characterized by the combination of kidney and eye inflammation in the absence of any other systemic or infectious causes otherwise explaining both pathologies. Uveitis may temporally coincide with TIN, but onset less than 2 months before or 14 months after kidney manifestations are common. The underlying etiopathogenic mechanisms of TINU syndrome are poorly understood, but it is thought to be an immune-mediated process in genetically susceptible individuals, triggered frequently by drugs or infections, although in many cases no cause can be identified.

Since the recent emergence of novel severe acute respiratory syndrome coronavirus 2 (SARS-CoV-2), publications have pointed to a special kidney tropism of this virus [1], with acute kidney injury (AKI) presentation as the main clinical scenario linked to the kidney damage caused by SARS-CoV-2. However, very few cases have been reported with TIN in adults [2, 3], with only two previously published pediatric cases related to SARS-CoV-2 infection and confirmation of viral presence in the kidney tissue in one of them [4, 5]. To our knowledge, this virus has not been implicated in TINU syndrome so far.

We report the case of a female adolescent with clinical manifestations compatible with TINU syndrome, where the most remarkable finding was the identification of SARS-CoV-2 in the kidney biopsies performed a few weeks and 10 months after the onset of symptoms.

## Case report

A previously healthy 12-year-old girl presented an elevation of serum creatinine (1.17 mg/dl), hypophosphatemia (2.7 mg/dl), hypouricemia (2.0 mg/dl), and ferritin elevation (279 ng/ml) identified in the context of a clinical history of 4 weeks of evolution, consisting of asthenia, anorexia, abdominal pain, vomiting, and weight loss. These manifestations had started after a self-limited febrile respiratory infection, managed with symptomatic treatment with ibuprofen. The patient had performed two negative unknown brand SARS-CoV-2 antigen tests at home (first and sixth day of the respiratory symptoms). For a few weeks, she was monitored by her primary care pediatrician, confirming the persistence of the previously described data, including a progressive weight loss of up to 12.5 kg and preserved diuresis. After 8 weeks, the patient tested positive in the PCR for SARS-CoV-2 (> 8 log_10_ RNA copies/ml of Omicron variant; cutoff > 3 log_10_ RNA copies/ml), requested for mild dysphonia. She had received two doses of Pfizer COVID-19 vaccination 4 and 5 months prior to presentation.

When the patient was referred to our tertiary care center (10 weeks of follow-up), previously reported abnormalities were confirmed, associating normocytic anemia (hemoglobin 10.3 g/dl), elevation of erythrocyte sedimentation rate (89 mm/hour), glycosuria, and a marked urinary elevation of beta-2-microglobulin (55.740 mcg/l). No evidence of recent Epstein-Barr, cytomegalovirus, hepatitis B or C, or HIV infections were found by serological testing. Antibodies against SARS-CoV-2 were not requested at this time. TIN was suspected and, subsequently, she was admitted to the hospital and underwent a percutaneous kidney biopsy at week 12, identifying the presence of an interstitial inflammatory infiltrate with lymphocyte predominance, with abundant eosinophils and plasma cells, tubulitis, and negative immunohistochemistry staining for SARS-CoV-2. Ophthalmology exploration did not reveal any simultaneous anomaly. At this moment, although some biochemical parameters showed a slight spontaneous improvement, oral steroid treatment (1 mg/kg/day) was started and maintained for a total of 9 weeks in a tapering schedule.

**Fig. 1:**
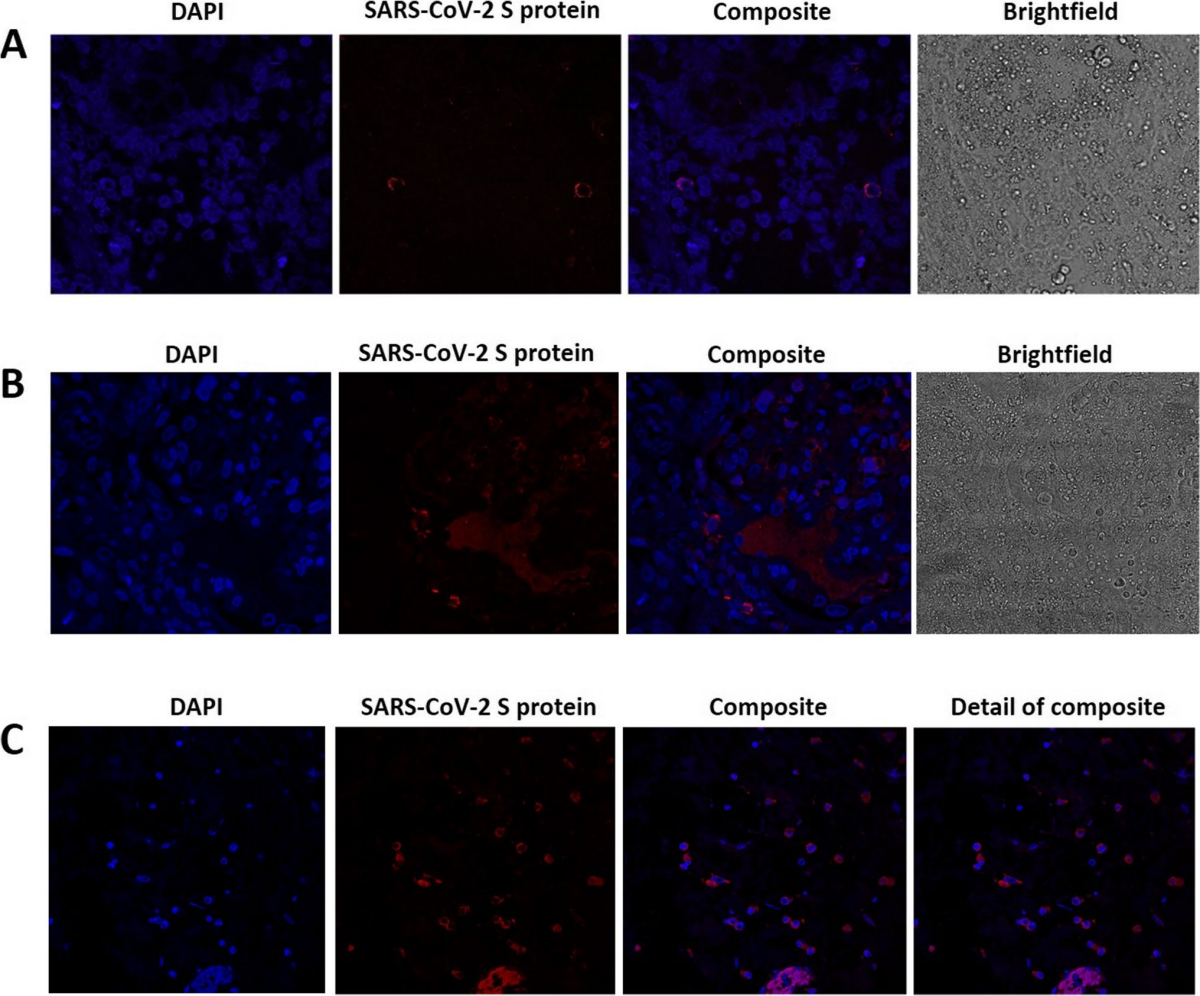
Immunofluorescence of kidney cells. Experiments with the first (**A**) and second (**B**) kidney biopsies (3 and 10 months following onset of clinical manifestations, respectively) are shown. The first column reveals cell nuclei using DAPI staining. In the second and third columns, SARS-CoV-2 S (spike) protein is shown in red, demonstrating a perinuclear pattern. A positive control spike-label slides from the intestine of the patient presented in the clinical case report by Mayordomo-Colunga et al. [6] is also included (**C**). Images were obtained by confocal microscopy (Leica SP8) and processed using ImageJ

To assess the presence of SARS-CoV-2 in the kidney, biopsy tissue slides were subjected to an immunofluorescence assay with a rabbit monoclonal antibody against SARS-CoV-2 spike protein (S protein), following the protocol described by Mayordomo-Colunga et al. [6], taking as positive control spike-label slides from the intestine of the patient presented in their clinical case. Immunofluorescence staining showed the presence of protein S in the intestine and within the kidney interstitium (Fig. 1).

**Fig. 2:**
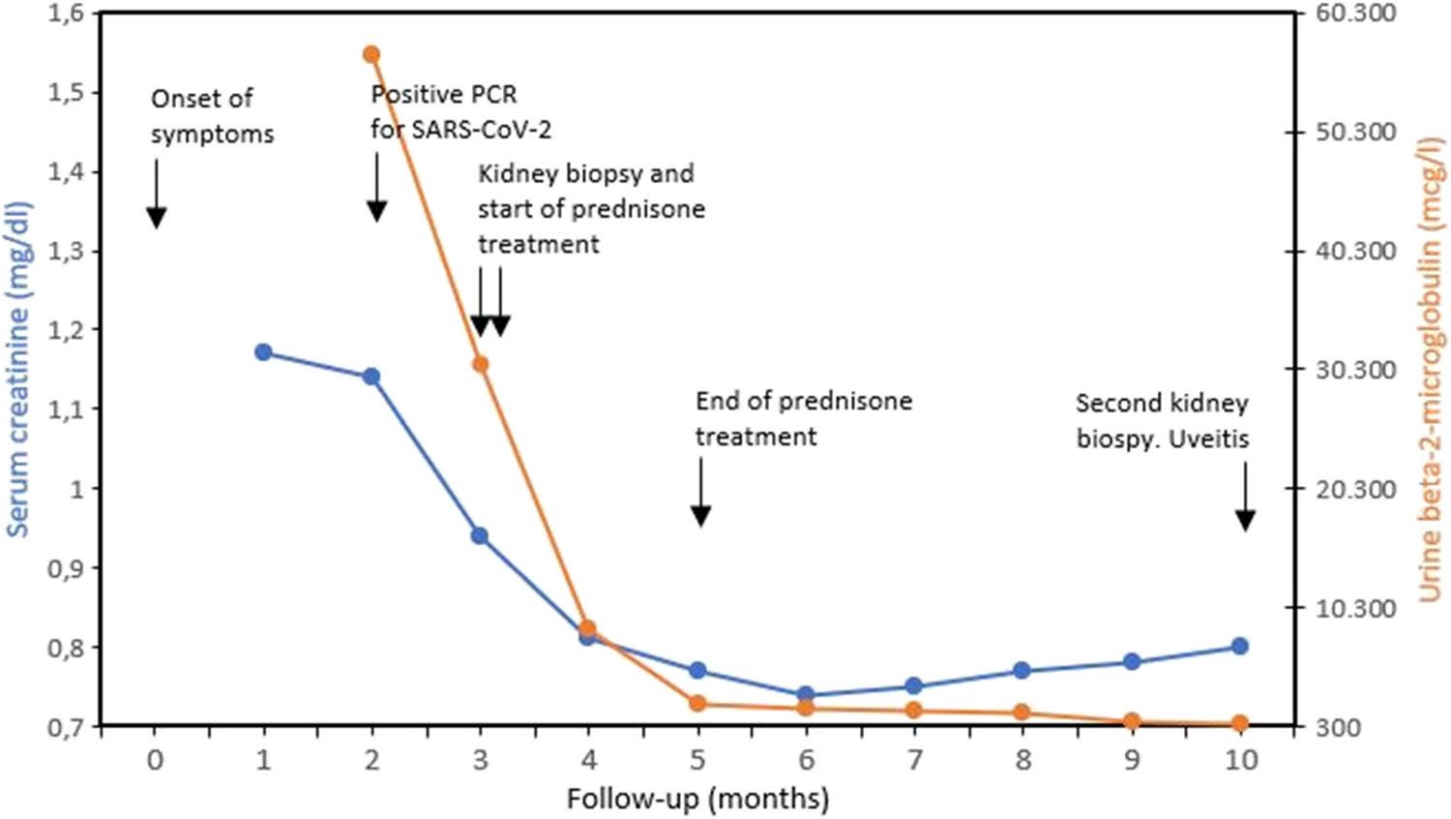
Time course of serum creatinine and urine beta-2-microglobulin over a 10-month period. The main clinical events are also indicated

During the following weeks, the patient was monitored, and a new ophthalmological examination ruled out ocular abnormalities. At the last clinic follow-up, ten months after clinical manifestations onset, serum creatinine and urine beta-2-microglobulin remained slightly elevated (0.80 mg/dl—eGFR 79 ml/min/1.73 m^2^—and 383 mcg/l, respectively) and kidney ultrasound showed mild bilateral parenchymal cortical thinning. A new percutaneous kidney biopsy was performed, showing absence of acute inflammation or chronic histological changes, but SARS-CoV-2 spike protein was again detected in kidney cells by immunofluorescence (Fig. 1). A simultaneous routine ophthalmological examination revealed asymptomatic bilateral anterior non-granulomatous uveitis. Further clinical investigation, including calcium metabolism, specific serological tests, parathyroid and thyroid-stimulating hormones, QuantiFERON-TB Gold Plus, antinuclear antibodies, anti-neutrophil cytoplasmic antibody, and HLA-B27 and HLA-B5 genotypes, excluded other infectious, inflammatory, or autoimmune diseases associating with ocular and kidney involvement. The time course of serum creatinine and urine beta-2-microglobulin since the onset of clinical manifestations is summarized in Fig. 2.

## Discussion

We describe a pediatric case with TIN and delayed uveitis, known as TINU syndrome, where SARS-CoV-2 spike protein within the kidney interstitium was identified in biopsies performed a few weeks and ten months following onset of clinical manifestations. To the best of our knowledge, the patient represents the first pediatric case with TINU syndrome where SARS-CoV-2 was detected in kidney tissue. The temporal sequence in our patient could suggest that TIN was initially triggered by ibuprofen intake or an infectious agent, and this certainly cannot be ruled out. However, the classically described infectious etiology is uncommon in both TIN or TINU syndrome (< 10% of cases reported), and the remarkable systemic manifestations or the absence of heavy proteinuria in the patient are not usual findings when ibuprofen participates in the pathogenesis of the clinical picture. Accordingly, our patient represents an idiopathic case of TINU syndrome, but the potential role of SARS-CoV-2 in kidney manifestations may be an attractive hypothesis.

As the initial symptoms coincided temporarily with a high load of infections during the COVID-19 pandemic (January 2021), the validity of the antigen tests carried out could be questioned. Although the sensitivity of these tests seems to be higher in respiratory symptomatic patients, it continues to be significantly lower than that of PCR tests [7, 8]. In addition, performing the antigen test at first and sixth days after the onset of the respiratory infection in our patient did not meet recently published recommendations [8]. Moreover, the risk of early reinfection by Omicron variant in individuals previously infected with other variants is significantly higher. This allows us to speculate on the possibility of another different SARS-CoV-2 variant originally infecting the patient [9], but obviously we cannot confirm it.

It is firmly established that SARS-CoV-2 has an undoubted potential for multi-organ damage, with the virus showing a characteristic kidney tropism [1]. Our patient presented early suggestive biochemical data of proximal tubular injury, with the presence of incomplete Fanconi syndrome. Although mild tubular involvement may appear secondary to other causes of TIN, this marked proximal tubular dysfunction appears to be a highly typical finding of SARS-CoV-2 infection, even in patients who are not critically ill [10]. The high abundance in proximal tubular cells of angiotensin-converting enzyme 2 (virus cell anchoring protein), may be one of the pathophysiological mechanisms that explain this association, especially demonstrated with the Omicron variant.

The most important link between SARS-CoV-2 infection and TINU syndrome in our patient depends on the objective identification of the virus in inflammatory cells of the kidney interstitium, and it could support a potential role in the associated manifestations and clinical evolution itself. As previously indicated, this patient would be the second case of TIN in a pediatric patient where SARS-CoV-2 has been detected in the kidney [4], with no other published cases showing kidney SARS-CoV-2 identification in cases with TINU syndrome. Both indirect and non-specific mechanisms are commonly involved in the injury associated with SARS-CoV-2 infection, but it has also been well established that the presence of SARS-CoV-2 in the kidney could be related to local damage, both due to direct cytopathic damage of the virus itself and secondarily linked to pro-inflammatory immunological mechanisms [11, 12]. Nearly all the virus infections may induce systemic immune response causing multiple tissue damage, but not all the viruses can directly invade the kidney. So far today, there is no evidence that respiratory viruses other than SARS-CoV-2 can do it, including in asymptomatic individuals or with mild infections [13].

Although the time delay between the onset of clinical presentation and the first kidney biopsy in our patient may seem too long (12 weeks), it is not very far from the case reported by Azukaitis et al. [4], who were able to demonstrate SARS-CoV-2 glycoprotein S by immunohistochemistry staining in the inflammatory cell infiltrate within kidney interstitium 6–7 weeks after the first manifestations. Therefore, we believe that this interval would not reject a potential role of SAR-CoV-2 in the demonstrated TIN either, as persistent kidney invasion by the virus has been recently shown by long-term viral replication in various body organs, including the kidney, even for more than 7 months after the original infection [14].

The patient has been followed-up for 10 months, and the last pathological clinical findings (serum creatinine, urine beta-2-microglobulin, and kidney ultrasound) were not exceptionally unexpected. Although the risk of chronic kidney disease induced by different causes of TIN is not negligible, recent published studies have shown that patients suffering COVID-19 exhibit increased risk of kidney outcomes (eGFR decline, kidney failure, major adverse kidney events, or steeper longitudinal decline in eGFR) in the post-acute phase of the disease, with SARS-CoV-2 probably contributing to profibrotic signaling in the kidneys, among other mechanisms [15]. The absence of significant histological findings in the second kidney biopsy performed on the patient does not allow us to explain the biochemical and sonographic abnormalities identified, but the sustained presence of SARS-CoV-2 particles in kidney tissue would not allow us to rule out its potential involvement in associated kidney damage.

In summary, we report an adolescent with clinical manifestations consistent with TINU syndrome of unknown cause and presence of SARS-CoV-2 in kidney tissue. Although direct causal relationship could not be demonstrated between SARS-CoV-2 and TINU syndrome, we hypothesize that SARS-CoV-2 was potentially involved in some way in this scenario, at least favoring or exacerbating the kidney injury and subsequent clinical symptoms. If this assertion is correct, our patient would be the first reported pediatric case of TINU syndrome where viral particles of SARS-CoV-2 could be isolated in kidney tissue. The persistence of some biochemical and ultrasound abnormalities and the demonstration of SARS-CoV-2 in the kidney after several months of evolution require long-term follow-up to rule out an unfavorable outcome. Subsequent investigations would be able to provide a better description of this rare complication and to know its prognosis in the medium and long term, including the risk of chronic kidney disease in the pediatric population.

## Data Availability

Data available on request due to privacy/ethical restrictions.
